# Proteomic Atlas of Notochordal Cell‐Derived Extracellular Vesicles Highlights EV‐Specific NF‐κB Modulation and Functional Implications of the Protein Corona

**DOI:** 10.1002/jex2.70186

**Published:** 2026-09-13

**Authors:** Josette C. van Maanen, Chantal Voskamp, Frank M. Riemers, Harmjan R. Vos, Anouk Eversdijk, Marije Kleinjan, Menno Bergmeijer, Martijn J. C. van Herwijnen, Stuart C. Howes, Frances C. Bach, Tim J. M. Welting, Christine L. Le Maitre, Guus G. H. van den Akker, Marca H. M. Wauben, Mariana A. Tryfonidou

**Affiliations:** ^1^ Department of Clinical Sciences Faculty of Veterinary Medicine Utrecht University Utrecht the Netherlands; ^2^ Center for Molecular Medicine University Medical Center Utrecht Utrecht the Netherlands; ^3^ Utrecht University, Oncode Institute Utrecht the Netherlands; ^4^ Department of Biomolecular Health Sciences Faculty of Veterinary Medicine Utrecht University Utrecht the Netherlands; ^5^ Structural Biochemistry, Bijvoet Center for Biomolecular Research Utrecht University Utrecht the Netherlands; ^6^ Laboratory for Experimental Orthopedics Department of Orthopedic Surgery, CAPHRI School for Public Health and Primary Care Maastricht University Maastricht the Netherlands; ^7^ Laboratory for Experimental Orthopaedics Department of Orthopaedic Surgery Maastricht University Medical Center+ Maastricht the Netherlands; ^8^ Division of Clinical Medicine Faculty of Health University of Sheffield Sheffield UK

**Keywords:** biomolecular corona, cartilage, exosomes, intervertebral disc, microvesicles, regeneration

## Abstract

The notochord is a central signalling hub during embryonic development. After regression of the notochord, notochordal cells (NCs) reside within the developing intervertebral disc. The NC‐secretome, including matrix, proteins and extracellular vesicles (NC‐EVs), promotes regeneration of degenerate intervertebral discs and cartilage. This study aims to unravel the NC‐EV‐associated proteome and modulatory role in regenerative processes. NC‐EVs were isolated from conditioned medium from porcine NC‐rich tissue using differential centrifugation, size exclusion chromatography and density gradient centrifugation. Proteomic analysis of NC‐EVs was performed using LC‐MS/MS and identified 4042 unique proteins, including EV‐associated markers, matrisome proteins and proteins associated with critical nodes in canonical signalling pathways. To test NC‐EV bioactivity, functional analysis was performed using SBE, TCF/LEF and NF‐κB luciferase reporters. NC‐EVs activated SMAD3 and inhibited IL‐1β‐mediated NF‐κB signalling. NC‐EV depleted controls showed similar effects, indicating that co‐isolated proteins played a major role in modulating signalling. Therefore, NC‐EVs were further purified and the GAG‐rich biomolecular corona was modified with hyaluronidase. Post‐hyaluronidase treatment, SMAD3 activation was enhanced only in methodological controls, while inhibition of IL‐1β‐mediated NF‐κB signalling was NC‐EV specific. This study is the first to reveal the NC‐EV proteome and illustrates that proteins absorbed on the NC‐EVs attribute to EV‐mediated functional effects.

## 1 Introduction

Notochordal cells (NCs) have received considerable attention in intervertebral disc (IVD) regeneration and, more recently, in cartilage regeneration (Bach et al., 2021). NCs are large vacuolated cells that reside in the notochord, a unique tissue that forms a mechanical scaffold in the developing embryo and directs differentiation of surrounding tissues (Choi and Harfe, 2011). Following notochord regression, NCs persist in the nucleus pulposus (NP) of the developing IVD, while retaining their signalling capacity (Rodrigues‐Pinto et al., 2016). In humans, these NCs are lost during IVD maturation and are absent in adult IVDs (Bach et al., 2021).

The NC‐secretome exerts regenerative effects on mature non‐vacuolated cells within the core of the IVD (NP cells) and on articular chondrocytes (Bach et al., 2021). In NP cells, it promotes proliferation, extracellular matrix (ECM) production and exerts anti‐inflammatory effects (Bach et al., 2021). Most studies use the porcine NC‐secretome, as NC‐rich pig NP tissue is readily available and has superior regenerative capacity, demonstrated by increased GAG production (Bach et al., 2015). The porcine NC‐secretome also increases GAGs and collagen type II production and reduces the production of catabolic markers such as MMP‐3, COX‐2, MMP‐13, IL‐6 and IL‐8 in chondrocytes derived from osteoarthritic cartilage (Müller et al., 2016). Furthermore, it induces mesenchymal stromal cell differentiation into a chondrocyte‐like phenotype with associated ECM production (Korecki et al., 2010, Purmessur et al., 2011). Taken together, these results indicate that the NC‐secretome mediates regenerative effects in cells with chondrocytic characteristics.

The NC‐secretome elicits biologic effects via extracellular vesicles (NC‐EVs) (Bach et al., 2016, 2017), small phospholipid bilayer‐enclosed particles released by cells, and the matrisome (Naba et al., 2012), composed of ECM proteins and ECM associated factors (including collagens, proteoglycans, glycoproteins, ECM regulators, ECM affiliated proteins and secreted factors) (de Vries et al., 2019; Laagland et al., 2023). NC‐EVs increase ECM deposition in a pellet culture of human and dog NP cells derived from degenerated discs (Bach et al., 2017), but fail to rescue the catabolic effects induced by IL‐1β (van Maanen et al., 2025). Mild immunomodulatory effects of NC‐EVs were observed in human patient‐derived NP tissues stimulated with IL‐1β, where NC‐EVs inhibited the secretion of IL‐6 and CXCL1 (Clos‐Sansalvador et al., 2022). Altogether, this highlights that potential regenerative effects of the NC‐secretome are, in part, driven by NC‐EVs. However, the pathways through which NC‐EVs modulate their anabolic and anti‐catabolic processes remain unknown. Therefore, in this study pathways associated with ECM regulation, including TGFβ (Chen et al., 2019; van der Kraan, 2018), and WNT (Gill et al., 2023; Wu et al., 2021), as well as inflammation‐related pathways such as IL‐1 and NF‐κB (Gill et al., 2023; Wu et al., 2021), were selected for further investigation. By examining downstream pathway activation (TGFβ and WNT) or inactivation (NF‐κB) upon NC‐EV stimulation, we aimed to better understand the mode of action underlying the biological effects of the NC‐EVs.

Within the NP tissue context, secreted NC‐EVs encounter a complex matrisome‐rich extracellular milieu (Hayes et al., 2001). These matrisome molecules can adsorb on EV surfaces and become part of the biomolecular corona. Thereby, EVs can act as scaffolds for the recruitment and binding of specific proteins, leading to downstream biological effects on recipient cells (Monopoli et al., 2012). These matrisome proteins may exert their downstream effects either through EV‐intrinsic signalling by modulating EV‐associated cellular uptake (Liam‐Or et al., 2024), or via surface‐mediated signalling by receptor‐ligand interactions (Lara et al., 2017). Given the fact that the NC‐derived matrix modulates anabolic and anti‐inflammatory signalling in both articular chondrocytes and NP cells (Bach et al., 2018; de Vries et al., 2018; Laagland et al., 2023), matrisome proteins associated to NC‐EVs may contribute to the regenerative properties of NC‐EVs. In line with this, the NC‐secretome is rich in GAGs, and NC‐EV preparations isolated from NC‐conditioned media through size exclusion chromatography (SEC) also contain GAGs (Bach et al., 2017). GAGs function not only as structural components of the core matrisome, but also as regulators of biological processes through interactions with growth factors, chemokines, cytokines and enzymes (Vallet et al., 2021). Which matrisome proteins are associated with NC‐EVs, as well as their relative contribution to downstream activation, remain elusive.

In this study, we performed mass spectrometry on isolated NC‐EVs to provide a detailed porcine NC‐EV protein atlas, and to identify NC‐EV‐associated proteins that could be linked to their regenerative and anti‐inflammatory effects. We assessed the NC‐EV signalling effects in selected pathways using a chondrocytic reporter cell line with specific transcriptional binding elements driving luciferase reporter gene expression (Housmans et al., 2023). We confirmed the influence of the biomolecular corona on NC‐EV signalling by conducting studies in reporter cells after depletion of the GAG‐rich biomolecular corona.

## 2 Materials and Methods

### 2.1 Isolation of NC‐EVs From Conditioned media

Seven complete porcine spines (3 months old) were collected from the local abattoir (Asten, NL) in accordance with national regulations. Each spine was processed separately to generate seven biological replicates (*N* = 7). Conditioned medium (NCCM) was generated from these healthy NC‐rich NP tissues (Lee et al., 2021) as described previously (Bach et al., 2015). To enrich for NC‐EVs, differential centrifugation was used to remove cells and debris, the supernatant was concentrated five times using a 3 kDa Pierce protein concentration tube (ThermoFisher, 88526), and aliquoted in low protein binding tubes (Thermo Scientific, 90411) and stored at ‐80°C until further use. Thereafter, different isolation techniques (Figure S1) were used, as described in detail in Supplementary File 1. In all functional experiments, EV‐depleted medium served as a procedural control to assess whether the observed effects are EV‐mediated and not related to co‐isolated proteins that contribute to the soft biomolecular corona. All samples were stored at ‐80°C in low protein binding tubes until further use. NC‐EVs from the same pig were used across all experiments, to improve comparability between datasets and strengthen the translatability of the findings. Due to limited sample availability, the final proof‐of‐concept experiment was performed using a pool of biological replicates. While pooling limits the assessment of inter‐donor variability, the pooled sample may better reflect the average biological response of the donor population, thereby increasing the representativeness of the results.

### 2.2 Size Exclusion Chromatography (SEC) With Commercial Columns for Functional Studies

Targeted functional studies were conducted with NC‐EVs isolated using qEV SEC‐columns (iZON Science) and pooling fractions 7–13 per pig spine. These fractions were based on the characterization of EV markers in previous work (van Maanen et al., 2023), and the measured protein concentrations. For the EV‐depleted procedural controls used in the functional experiments, these EV‐enriched samples were ultracentrifuged at 100,000 *g* for 15–18 h at 4°C, whereafter the supernatant was collected and stored.

### 2.3 SEC and Sucrose Density Gradient Centrifugation and Mass Spectrometry

EV‐enriched samples derived from 6 SEC columns per pig spine were pooled, pelleted at 100,000 *g*, and subjected to linear sucrose density gradients (2.0–0.4 M sucrose). The EV‐containing fractions (1.09–1.16 g/mL densities (Bach et al., 2017)) were pooled per pig spine, and pelleted at 100,000 *g*. These pellets were resuspended in PBS (NC‐EV sample) and stored until liquid chromatography‐tandem mass spectrometry (LC‐MS/MS) was performed on seven biological replicates. Following tryptic digestion, peptides were desalted using C18 stage tips and separated by reversed‐phase nanoLC on a 25 cm in‐house packed C18 column using a 240‐min acetonitrile gradient. Peptides were analysed on an Orbitrap Eclipse mass spectrometer coupled to an Easy‐nLC 1200 system and operated in data‐dependent acquisition mode with FAIMS. Full MS scans were acquired at high resolution, followed by HCD fragmentation of the most abundant precursor ions.

### 2.4 Iodixanol Density Gradient Centrifugation and SEC for Validation Functional Studies

To confirm the functional NC‐EV effects, we repeated key functional experiments using a 60%–10% iodixanol gradient (Optiprep, Progen Biotechnik GmbH) which is a more stringent purification method. In addition, hyaluronidase treatment (Boere et al., 2016) prior to purification was used to hydrolyse GAGs from the NC‐EV biomolecular corona. Due to limited sample availability, this set of experiments was performed using a pooled sample. OptiPrep gradients were ultracentrifuged at 192,000 *g* for 15–18 h at 4°C, whereafter densities of 1.06–1.19 g/mL were collected, pooled and stored. To generate a NC‐EV‐depleted methodological control, the supernatant of the initial OptiPrep gradient was collected and loaded onto a new OptiPrep gradient whereafter the same fractions were collected (Figure 6A). For functional analysis, OptiPrep was replaced with HgDMEM using a SEC column (20 mL; Bio‐Rad Laboratories) with 15 mL Sephadex G‐100 (Sigma‐Aldrich). The seven fractions containing NC‐EVs (between fractions 3–9, each 1 mL) were pooled and stored.

### 2.5 Mass Spectrometry Data Processing

Raw mass spectrometry data were analysed using Proteome Discoverer 2.5 against the UniProt *Sus scrofa* database (December 2023). Protein abundance data were further processed in Perseus (v2.10.0). Only proteins identified with at least three peptides, including two unique peptides, were included. Data were log_2_‐transformed, and proteins consistently detected across EV samples were used for downstream analysis. Proteins identified in at least four NC‐EV samples were analysed using pathway databases in KEGG in the period April‐August 2025. This set was further enriched with NC‐EV proteins that could either act as stimulators and/or inhibitors using Uniprot and STRING (version 12.0) databases and corroborated with literature research using PubMed. To relate the identified NC‐EVs proteins to biological processes, a functional enrichment analysis using g:GOst was performed (Reimand et al., 2007) and using Gene Ontology (Ashburner et al., 2000) and Reactome (Griss et al., 2020) databases. Next, we clustered the individual enrichment terms into defined categories based on functional and protein overlap.

### 2.6 Nanoparticle Tracking Analysis (NTA)

Particle concentration and size distribution of NC‐EVs and EV‐depleted samples were determined by nanoparticle tracking analysis (NTA) using a NanoSight NS500 (Malvern Panalytical). Samples were diluted in Dulbecco's Phosphate Buffered Saline and measured using NTA software v3.4. For each sample, five 30s videos were recorded at 25°C and analysed.

### 2.7 Cryo‐electron Microscopy

Holey carbon R2/1 (Quantifoil Micro Tools) copper 200‐mesh EM grids were glow discharged at 0.39 mbar with 15 mA for 25 s using a PELCO easiGlow discharger (Ted Pella). 20 µL of isolated NC‐EV sample was placed on ice‐cooled parafilm for about 1 min. With the carbon side facing down, grids were incubated on the droplet for approximately 1 min and taken to a Vitrobot Mark IV (Thermo Fisher Scientific) and blotted for 3.5 s using filter paper (Whatman no. 1) at blot force ‐5 with the environmental chamber set to 21°C and 95% relative humidity and immediately plunged into pure ethane. EM grids were clipped into autogrid cartridges (Thermo Fisher Scientific) and stored under liquid nitrogen until imaging.

Localization and imaging of EVs was performed using a Talos Arctica TEM (Thermo Fisher Scientific) operated at 200 keV and equipped with a post‐column energy filter and a K2 detector (Gatan). For localisation, medium magnification tilesets were captured at a pixel size of 18.9 Å at specimen level. Next, individual EVs or groups of them were imaged using a pixel size of 2.2 Å and a total dose of 26 e/Å^2^ in a 6 s exposure. An optimised target defocus of –3 µm and an energy filter slit‐width of 20 eV helped to ensure the contrast was sufficient to distinguish the lipid bilayers. All images were captured in SerialEM Version 4.1.22 (Mastronarde, 2005). Images were reviewed and figures prepared using IMOD (Mastronarde and Held, 2017).

### 2.8 Functional Pathway Validation Using Stable Reporter Cell Lines

SW1353 reporter cell lines, which have been instrumental as an in vitro OA model (Pang et al., 2021), were seeded (33,000 cells/cm^2^) into 384‐well plates, and serum‐starved overnight (HgDMEM + 0.5% EV‐depleted FBS + 1% P/S) to reduce background reporter signalling while maintaining viability. Thereafter, cells were washed twice (HgDMEM + 0.1% EV‐depleted FBS + 1% P/S) and stimulated for 6 h. To study the EV‐specific inhibitory effect on the NF‐κB reporter, all media were supplemented with 0.1 ng/mL IL‐1β (SRP3083, Sigma), while the EV‐specific activation on the SBE and TCF/LEF reporter was studied in the absence of other positive stimuli (Housmans et al., 2023). After stimulation, the cells were washed, lysed with MilliQ and nanoLuc luciferase activity was measured by adding Nano‐Glo (N1120, Promega). Luminescence was measured with a Tristar2 LB942 multi‐mode plate reader (Berthold Technologies) at room temperature. The fold change was calculated after correcting the Relative Light Units (RLU) for the blank measurement and dividing that by the RLU of the unstimulated control.

### 2.9 Statistical Analysis

Data was analysed in GraphPad Prism 10.4.1. Data were examined for normal and log normal distribution D'Agostino & Pearson test. All data were normally distributed. For NTA data (unequal variances), a Brown‐Forsythe ANOVA was performed, followed by a Dunnett T3 post hoc test. For the reporter data, a one‐way ANOVA was performed followed with a Šídák's post hoc test. For the reporter data of NC‐EVs isolated with the OptiPrep gradient, a one‐way ANOVA was performed followed by a Fisher's Least Significant Difference (LSD) test; correction for multiple comparisons was not applied given the homeostatic modulatory role of the relatively pure EV‐preparation, relatively small sample size and to minimise the likelihood of overlooking biological meaningful trends. In all tests, *p*‐value < 0.05 was considered significant.

## 3 Results

### 3.1 Isolation and Characterization of Porcine NC‐EV

**FIGURE 1:**
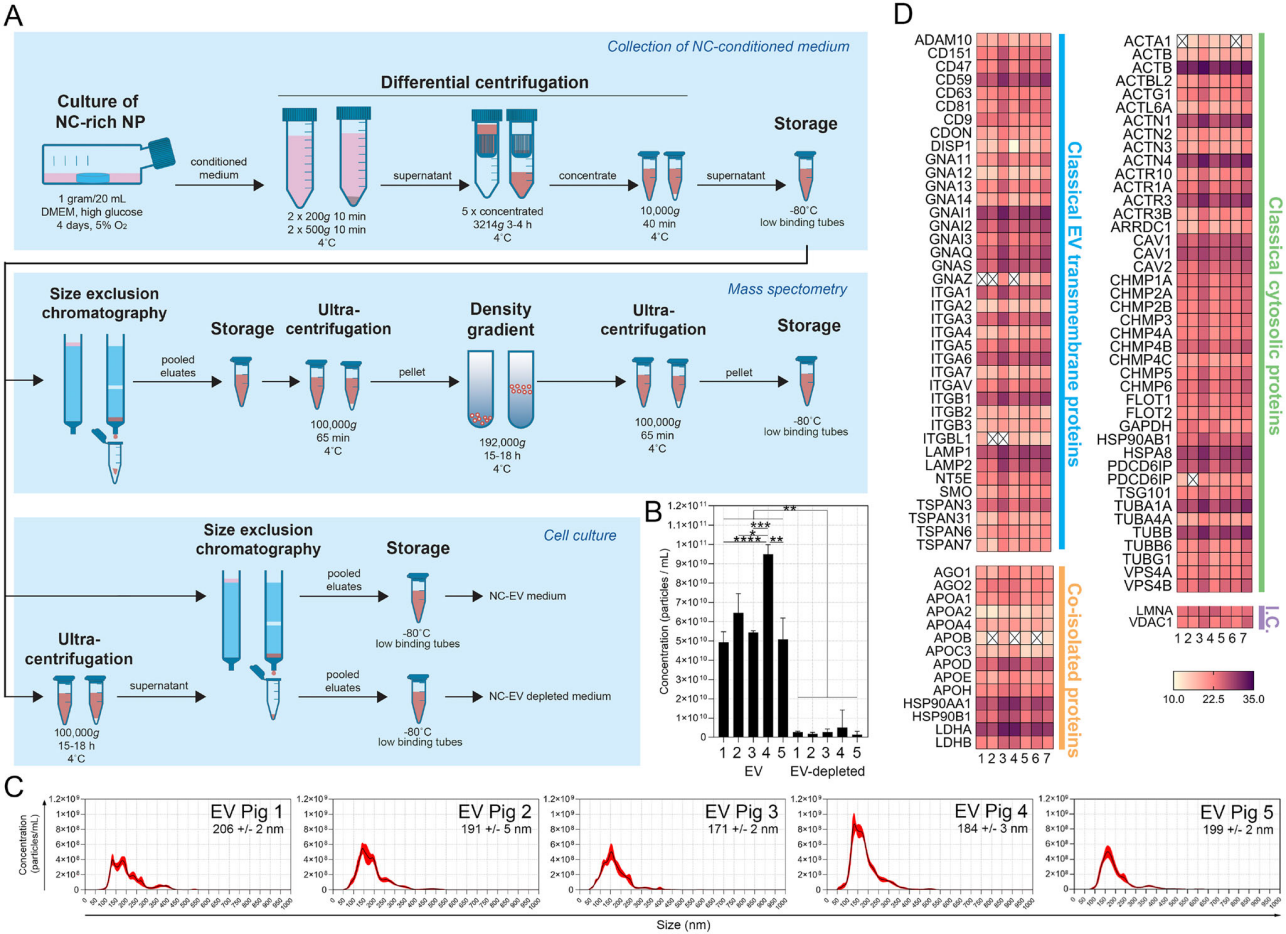
Isolation and characterisation of porcine NC‐EV (A) Schematic representation of the isolation and purification steps before mass spectrometry and cell culture. Notochordal cell (NC) conditioned medium was generated by culturing porcine NC‐rich nucleus pulposus (NP) tissue. Differential centrifugation removes cells and apoptotic bodies, and size exclusion chromatography (SEC) removes larger protein complexes. For mass spectrometry, NC‐EVs were isolated from the SEC medium by ultracentrifugation and the 100,000 g pellet was purified on a linear sucrose gradient and pelleted again by ultracentrifugation. For cell culture, the SEC isolated medium was directly used (NC‐EV medium) or depleted of NC‐EVs by ultracentrifugation (NC‐EV‐depleted medium). (B–C) Particle concentration (B) and size distribution (C) of isolated NC‐EVs and their respective EV‐depleted controls from five different porcine spines using NanoSight NS500. Graphs show the mean and SD of five captures of 30 s. (D) Heat map of EV markers in the NC‐EV proteome shows mean log_2_ abundance (range 10–35). *N* = 7 porcine spines.

To collect enriched and purified NC‐EVs from NCCM for proteomic analysis, a combination of differential centrifugation, SEC and sucrose density gradient flotation was done, while only differential centrifugation and SEC was performed for functional experiments (Figure 1A, Figure S1, Figure S2A). Furthermore, for functional experiments, EV‐depleted medium served as a procedural control to study whether the observed effects are EV‐mediated and not related to co‐isolated proteins contributing to the soft biomolecular corona. The particle concentration and size was quantified using NTA (mean 6.3 × 10^10^ ± 1.9 × 10^10^ particles/mL in NC‐EV samples; Figure 1B). The matched EV‐depleted samples had a lower concentration (2.7 × 10^9^ ± 1.4 × 10^9^ particles/mL) and matched closer the particle background concentration of PBS (2.5 × 10^7^ particles/mL). Based on the NTA results, we estimated the particle concentration per gram tissue for NC‐EV (1.3 × 10^12^ ± 3.8 × 10^11^ particles/gram NP) and EV‐depleted samples (5.4 × 10^10^ ± 2.9 × 10^10^ particles/gram NP). Little variation was observed between particle concentrations from different animals, however pig 4 had a significantly higher particle concentration compared to the others. The average size of the NC‐EV particles was 190 ± 13 nm with little variation between samples (Figure 1C), whilst the EV‐depleted sample showed a less defined size profile (Figure S2B).

Proteomic analysis of these purified NC‐EVs, identifying 4042 unique proteins, of which 3657 were identified in all seven NC‐EV samples (Figure S2C). Hierarchical clustering based on the relative protein abundance showed similar profiles between NC‐EVs and was independent of the NC‐EV pig (Figure S2D). To ensure confidence, further analysis was performed on proteins that were identified in at least four pigs.

We analysed the presence of EV‐associated markers, following the guidelines for the minimal information for EV studies (MISEV2018 and MISEV2024) (Thery et al., 2018; Welsh et al., 2024). A large number of classical transmembrane proteins were present (category 1) such as tetraspanins, multi‐pass membrane proteins and integrins, as well as classical cytosolic proteins (category 2) including flotillins and caveolins (Figure 1D), confirming the presence of general EV features. Furthermore, we analysed the presence of co‐isolates (category 3) and found 14 proteins often presumed to be associated with non‐EV structures (Figure 1D) (Welsh et al., 2024). However, seeing the increasing awareness of the complexity of the biomolecular corona, and its role in EV biology (Esmaeili et al., 2025), we cannot exclude that these proteins might also be associated with NC‐EVs. Lastly, category 4 provides additional information on intracellular origins, and we found proteins associated with the cell nucleus (LMNA) and mitochondria (VDAC), indicating that the NC‐EVs contain components of the NC nucleus and mitochondria (Figure 1D). Detection of VDAC likely reflects co‐isolation of mitochondrial fragments, considering that a robust NC‐EV isolation protocol was applied, combining differential centrifugation, SEC and density gradient ultracentrifugation, which is expected to exclude crude mitochondria (Clos‐Sansalvador et al., 2022). Whether intact mitochondria or mitochondria‐derived compartments are associated with the NC‐EVs remains to be determined. Furthermore, we compared the NC‐EVs with EV studies present in Vesiclepedia, a manually curated database containing molecular data on a broad range of types of EVs and species (Chitti et al., 2024; Kalra et al., 2012; Pathan et al., 2019). Ninety‐six percent of NC‐EV proteins overlapped with Vesiclepedia proteins (version 6.1.25). There were 166 NC‐EV proteins that have not previously been reported, of which 59 (36%) were unidentified proteins in the less annotated pig proteome (Zhao et al., 2021). The remaining 107 uniquely identified NC‐EV proteins contained multiple different proteins, including TBXT, a protein involved in NC biology and absent in other tissues (Figure S3). Overall, this study presents a comprehensive NC‐EV proteomic data set based on 7 biological replicates containing EV‐associated markers, covering amongst others membrane proteins that are notoriously difficult to detect in proteomics.

## 4 NC‐EVs Contain a Large Number of Matrisome Proteins

**FIGURE 2:**
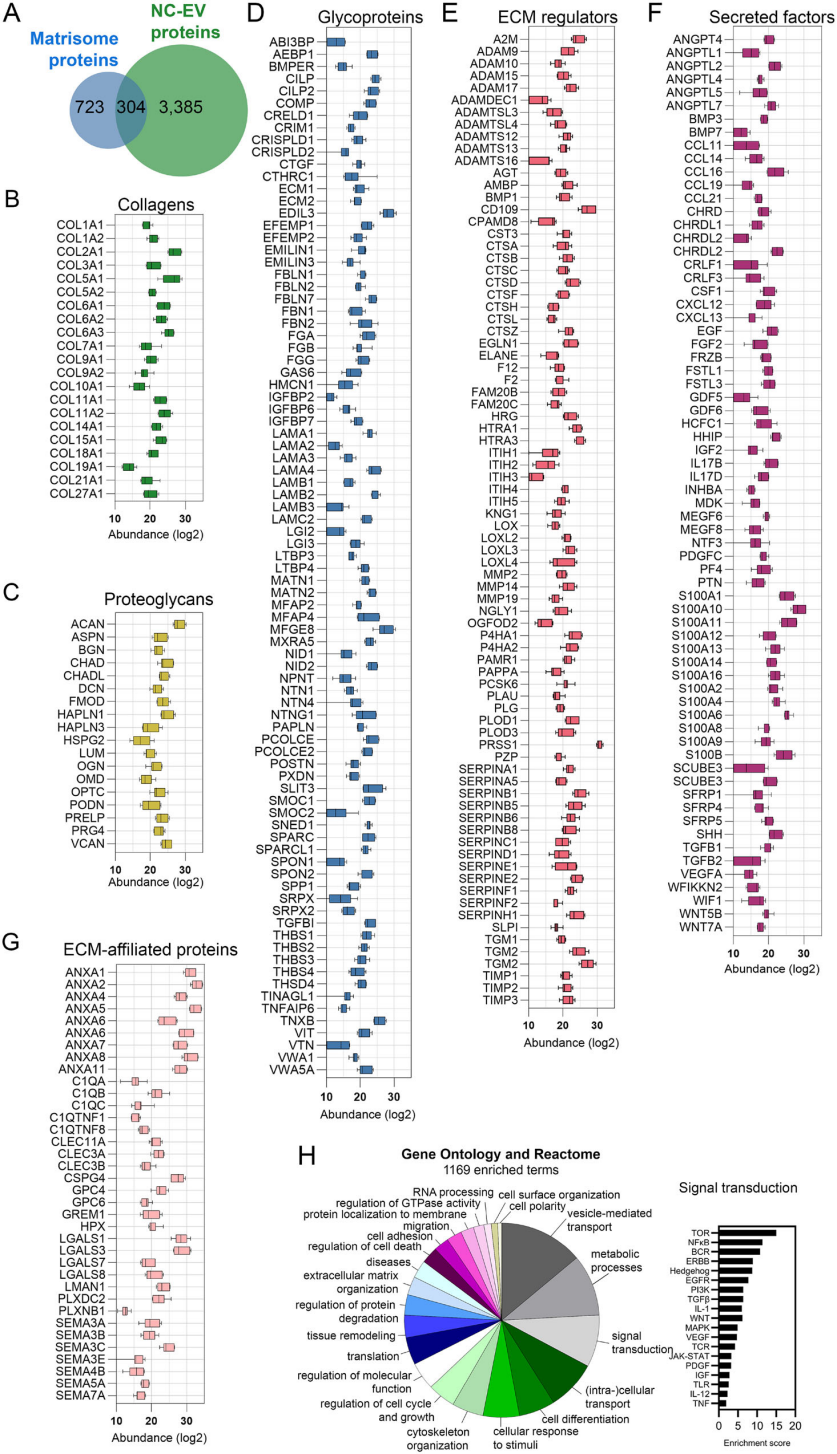
Functional annotation of NC‐EV proteome (A) Venn diagram shows overlap between matrisome proteins and NC‐EV proteome. (B–G) Mean log_2_ abundance of matrisome proteins present in the NC‐EV for each matrisome category. The log_2_ abundance ranges between 10 and 35, and for visualisation, missing data were imputed with a constant value of 10; *N* = 7 pig spines. (H) Pie chart showing the percentage of clustered Gene Ontology and Reactome terms of proteins identified in at least four spines. The terms were manually clustered based on functional overlap to facilitate clear graphical representation, with a side panel zooming in on the signal transduction pathways.

The IVD and cartilage are tissues rich in ECM, and therefore we focused on ECM‐related proteins, referred to as ‘the matrisome’ (Naba et al., 2012). Thirty percent of the matrisome proteins were detected in the NC‐EV proteome, and accounted for eight percent of the total NC‐EV proteome (Figure 2A; Supplementary Table 1). The top 10 most abundant core matrisome proteins were ACAN, EDIL3, MFGE8, COL2A1, COL5A1, TNXB, COL6A3, HAPLN1, VCAN and CILP (Figure 2B‐D). ANXA2, ANXA5, ANXA1, ANXA8, PRSS1, ANXA6, S100A10, LGALS3, LGALS1 and ANXA4 were the most abundant non‐core matrisome‐associated proteins (Figure 2E‐G). These results indicate that matrisome proteins are associated with NC‐EVs and, given the extensive purification steps used to isolate them, may be part of the EV protein corona.

## 5 Proteins Associated With NC‐EVs are Involved in a Wide Variety of Biological Processes

Functional enrichment analysis revealed significant enrichment of 1169 biological processes which were clustered into 22 categories based on functional and protein overlap (Figure 2H; Supplementary Table 2). To understand how the NC‐EV cargo affects signalling cascades involved in IVD and cartilage tissue regeneration, we focused on enrichment terms linked to signal transduction pathways. We identified 19 specific signalling pathways enriched in the NC‐EV cargo (Figure 2H), with most involved in the regulation of inflammation, while others could be linked to tissue remodelling.

Given that cartilage and IVD degeneration come with altered ECM deposition and uncontrolled inflammatory factors, further in‐depth analysis and functional studies focused on how NC‐EVs, including their biomolecular corona, could modulate TGFβ (Chen et al., 2019; van der Kraan, 2018), WNT (Gill et al., 2023; Wu et al., 2021), IL‐1 and NF‐κB (Gill et al., 2023; Wu et al., 2021) signalling. To understand and visualise at what level NC‐EV proteins are associated with these signalling pathways, in‐depth data analysis was conducted using protein‐protein interaction and pathway databases (KEGG, Reactome, UniProt and STRING), and corroborated with literature research (PubMed; Figure S4). To enable a clear visualisation and interpretation of the data, a summary of each pathway analysis is shown in the main figures with a network showing direction of stimulation/inhibition and a heatmap showing protein abundance. Functional analysis was conducted using NC‐EVs isolated by differential centrifugation and SEC, and their effects were assessed using chondrocyte‐like reporter cell lines, each with a specific transcriptional binding element driving luciferase expression. First, the interaction of SW1353 reporter cell lines with NC‐EVs was confirmed using MemGlow‐ and Maleimid‐labelled NC‐EVs (Figure S5). In the following paragraphs, each specific pathway will be explained in the light of the NC‐EV proteome.

## 6 NC‐EV Associated Proteins Orchestrate Distinct Signalling Cascades in SW1353 Recipient Cells

### 6.1 SMAD3 Signalling

**FIGURE 3:**
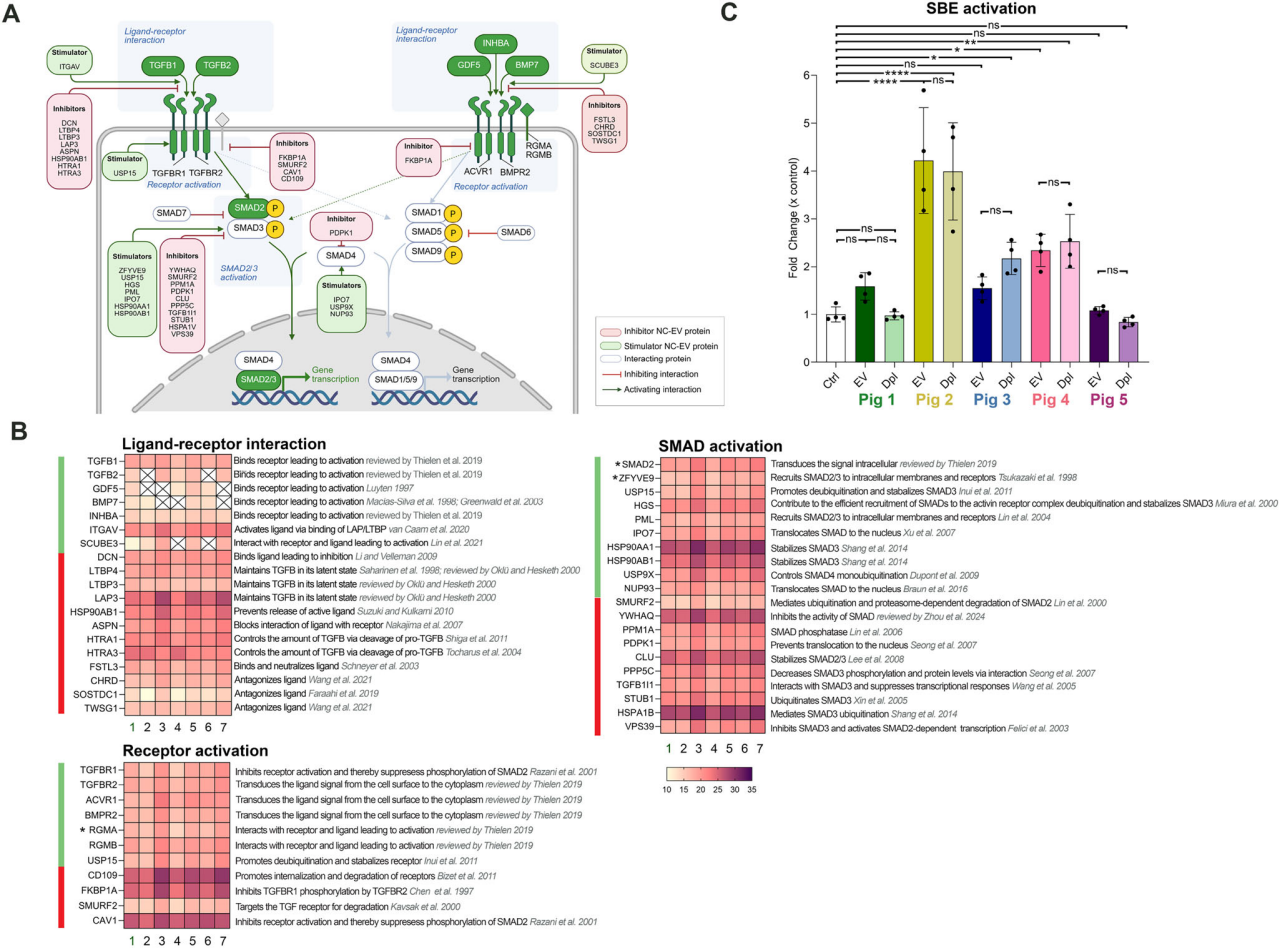
NC‐EVs and NC‐EV‐depleted samples enhance SMAD3 signalling in SW1353 cells (A) Simplified schematic representation of key proteins involved in canonical SMAD3 signalling. Red and green boxes indicate NC‐EV proteins that activate or inhibit SMAD3 signalling, respectively. White boxes are interacting proteins that were not detected in NC‐EV samples from at least four pig spines. The signalling pathway was curated based on current literature. (B) Heat map of selected proteins in the NC‐EV proteome shows mean log_2_ abundance (range 10–35). For each protein, the function related to the SMAD3 signalling pathway was noted based on current literature (green = activators; red = inhibitors). *N* = 7 pig spines. (C) Smad binding element (SBE) driven Nano luciferase. A stable reporter‐expressing SW1353 cell line was stimulated with either NC‐EVs or NC‐EV‐depleted controls for 6 h. As a control medium (Ctrl), SEC medium was used. Each condition (N = 5 biological replicates) was measured in quadruplet (technical replicates); data are displayed as the mean. Significance of differences between experimental groups was detected using one‐way ANOVA tests. *P ≤ 0.05; **P ≤ 0.01; ****P ≤ 0.0001; ns, nonsignificant.

To integrate the proteomic data analysis with biological understanding, we worked with a simplified representation of the SMAD3 pathway consisting of three functional nodes: (1) ligand‐receptor interaction, (2) receptor activation and (3) SMAD activation (Figure 3A). NC‐EV‐associated proteins are linked to all three functional nodes in the SMAD3 pathway, including 25 stimulators and 24 inhibitors with varying abundance (Figure 3B), indicating both activating and inhibitory effects. Given the reported anabolic NC‐EV effects on the matrix in cartilage (van der Kraan, 2018) and disc cells (Chen et al., 2019), we studied the effect of NC‐EVs on SMAD3 signalling using the SW1353 reporting SMAD3 activity (Smad binding element (SBE)‐reporter). Exposure of NC‐EVs for 6 h increased luciferase activity on average 2.1‐fold compared to control media. Statistical significance was, however, not consistent across all NC‐EVs biological replicates (4.2‐fold vs. 1.1‐fold) (Figure 3C). Similar patterns and comparable levels of activation were observed in the EV‐depleted group. These results suggest that the marked SMAD3 activation upon incubation with NC‐EVs and EV‐depleted controls from pigs 2 and 4 was most likely caused by secretome proteins that adsorb on the EV surface and become part of the soft protein corona.

### 6.2 Canonical Wnt Signalling

**FIGURE 4:**
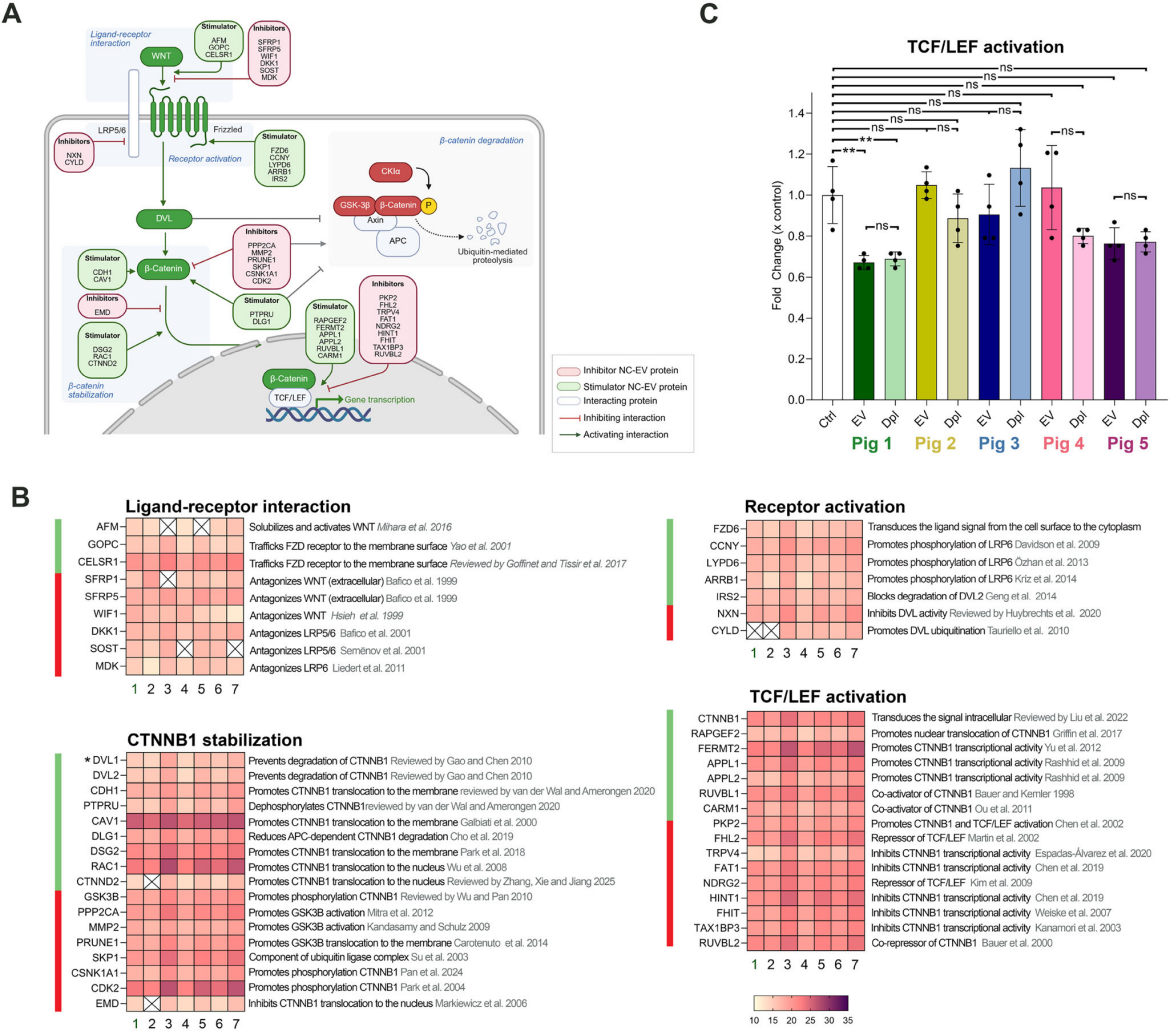
NC‐EVs and NC‐EV‐depleted samples do not activate TCF/LEF signalling in SW1353 cells (A) Simplified schematic representation of key proteins involved in canonical WNT signalling. Red and green boxes indicate NC‐EV proteins that activate or inhibit WNT signalling, respectively. White boxes are interacting proteins that were not detected in NC‐EV samples from at least four spines. The signalling pathway was curated based on current literature. (B) Heat map of selected proteins in the NC‐EV proteome shows mean log_2_ abundance (range 10–35). For each protein, the function related to the WNT signalling pathway was noted based on current literature (green = activators; red = inhibitors). *N* = 7 pig spines. (C) TCF/LEF driven Nano luciferase. A stable reporter‐expressing SW1353 cell line was stimulated with either NC‐EVs or NC‐EV‐depleted controls for 6 h. Each condition (*N* = 5 biological replicates) was measured in quadruplet (technical replicates); data are displayed as the mean. The control medium (Ctrl) SEC medium was used. Significance of differences between experimental groups was detected using one‐way ANOVA tests.**P ≤ 0.01; ns, nonsignificant.

We identified 25 stimulators and 25 inhibitors with varying abundance in the NC‐EV associated proteins around three functional nodes: (1) ligand‐receptor interaction, (2) receptor activation, (3) CTNNB1, gene encoding for β‐catenin, stabilization and (4) TCF/LEF activation (Figure 4A‐B). Given the known and context‐dependent effects of canonical WNT signalling at the matrix level of cartilaginous tissues (Gill et al., 2023; Wu et al., 2021), the stimulating effect of NC‐EVs on WNT signalling was determined using the SW1353 cell line reporting activity of TCF/LEF. Overall, NC‐EV incubation did not activate TCF/LEF compared to control media, nor did the EV‐depleted controls (Figure 4C).

### 6.3 NF‐ĸB Signalling

**FIGURE 5:**
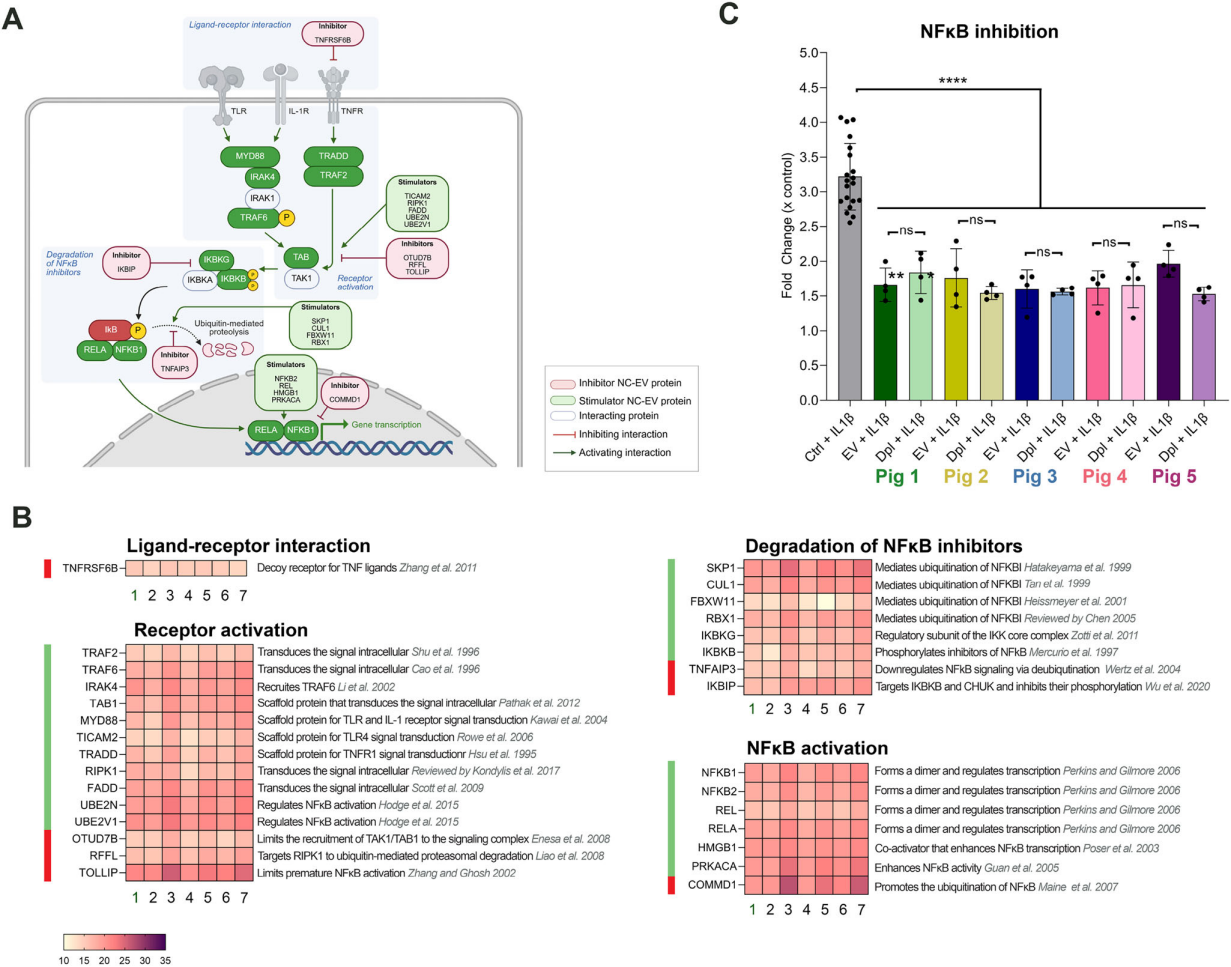
NC‐EV‐related proteins around NF‐κB signalling (A) Simplified schematic representation of key proteins involved in NF‐κB signalling. Red and green boxes indicate NC‐EV proteins that either activate or inhibit NF‐κB signalling, respectively. White boxes are interacting proteins that were not detected in NC‐EV samples from at least four spines. The signalling pathway was curated based on current literature. (B) Heat map of selected proteins in the NC‐EV proteome shows mean log_2_ abundance (range 10–35). For each protein, the function related to the NFκB signalling pathway was noted based on current literature (green = activators; red = inhibitors). *N* = 7 pig spines. (C) NF‐κB driven Nano luciferase. A stable reporter‐expressing SW1353 cell line was stimulated with either NC‐EVs or NC‐EV‐depleted controls for 6 h. Each condition (*N* = 5 biological replicates) was measured in quadruplet (technical replicates) in the presence of IL1β; data are displayed as the mean. As a control medium (Ctrl), SEC medium was used in the presence and absence of IL1β. Significance of differences between experimental groups was detected using one‐way ANOVA tests. ****P ≤ 0.0001; ns, nonsignificant.

The NC‐EV proteome contains several proteins that interact with protein hubs regulating NF‐ĸB signalling. A simplified representation of the canonical NF‐ĸB pathway consists of three functional nodes: (1) ligand‐receptor interaction, (2) receptor activation, (3) IκBα degradation (Inhibitor of kappa Bα, binds to NF‐ĸB and prevents nuclear translocation) and (4) NF‐κB activation (Figure 5A). NC‐EV‐associated proteins are connected to every functional node of the NF‐κB pathway, including 23 stimulators and 7 inhibitors with variable abundance (Figure 5B), suggesting both activating and inhibitory effects. Considering prior indications of the anti‐inflammatory properties of NC‐EVs (van Maanen et al., 2025), we determined the net NC‐EVs effect on NF‐κB signalling under inflammatory conditions in SW1353 reporting activity of the transcription factor binding elements of NF‐κB was tested in the presence of 100 pg/mL IL‐1β (Figure 5C). Exposure to NC‐EVs for six hours significantly reduced the luciferase activity in all cells with an average of 50% reduction compared to controls stimulated with IL‐1β. However, no difference was observed between the EV and EV‐depleted samples, suggesting that the effect was caused by proteins present in the protein corona of the NC‐EVs, rather than by intrinsic NC‐EV associated proteins.

## 7 NC‐EV Specific Effects on Distinct Signalling Cascades in SW1353 Recipient Cells and the Impact of Hyaluronidase Treatment

**FIGURE 6:**
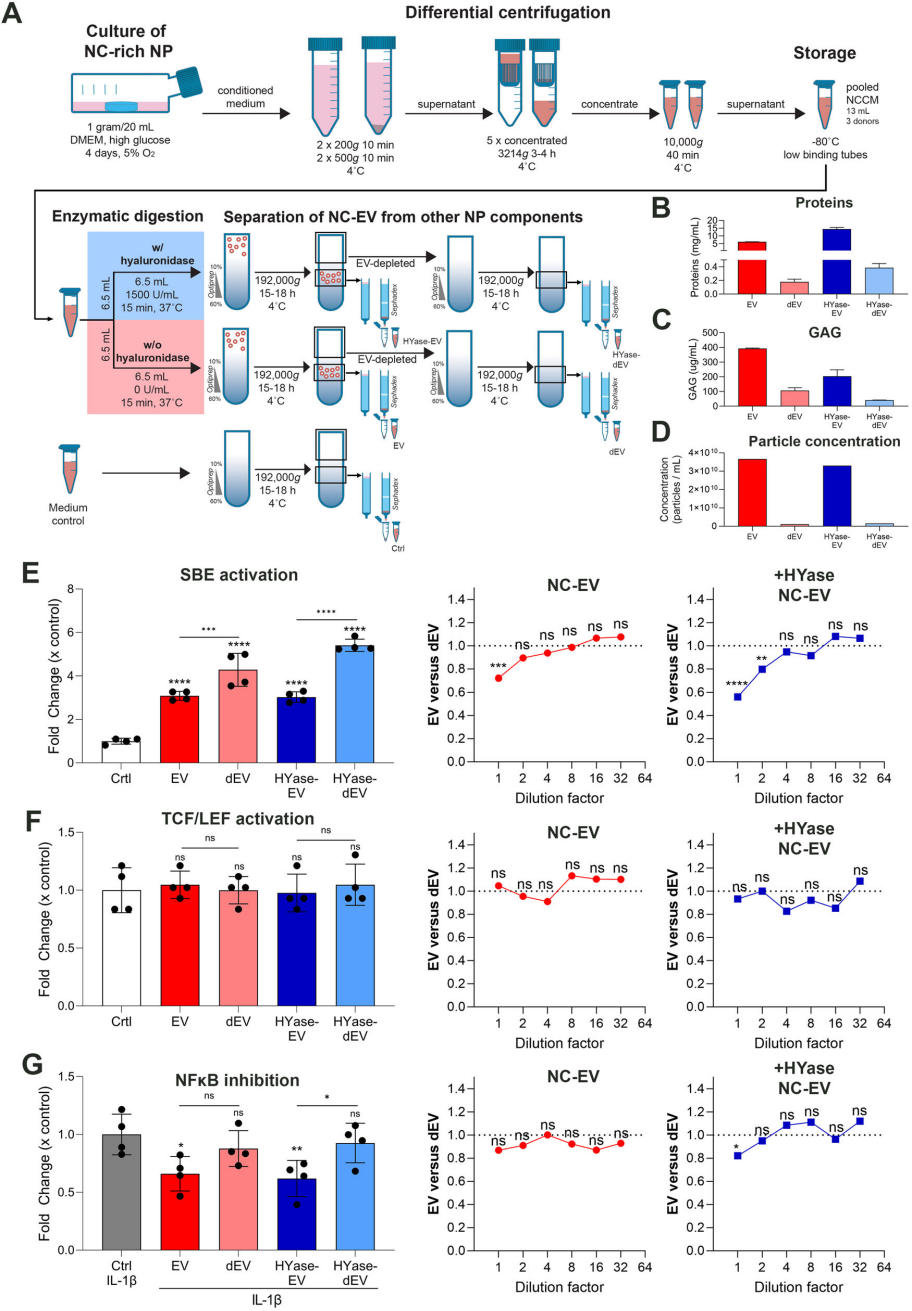
Reporter activity of SW1353 cell lines upon NC‐EVs that are isolated with OptiPrep, size exclusion chromatography, and hyaluronidase A. Schematic representation of the NC‐EV isolation procedure to study the function of the biomolecular corona. B‐D. NC‐EV characterization of protein (B), glycosaminoglycans (GAG) and particle (C) concentration. E‐G. SBE (E), TCF/LEF (F), and NF‐κB (G) driven Nano luciferase. A stable reporter‐expressing SW1353 cell line was stimulated with either NC‐EVs or NC‐EV‐depleted controls for 6 h. The right panel shows the dose effect of 2‐fold serial dilutions, showing a ratio between luciferase activity of NC‐EVs versus depleted NC‐EVs. Significance of differences between experimental groups was detected using one‐way ANOVA tests. *P ≤ 0.05; **P ≤ 0.01; ***P ≤ 0.001; ****P ≤ 0.0001; ns, nonsignificant.

To explore whether the protein corona governs NC‐EV function, NC‐EVs were isolated using an OptiPrep‐based purification method that yields a purer EV‐preparation than SEC alone, while allowing functional studies (Van Deun et al., 2014; Zonneveld et al., 2014, 2021). Furthermore, hyaluronidase was used to modify part of the GAG‐rich biomolecular corona from the NC‐EVs without altering the particle integrity, size and concentration (HYase‐EV) and resulted in a 3‐fold reduced GAGs/particles ratio (Figure 6A‐D; Figure S6A‐B). Cryogenic transmission electron microscopy images further confirmed that the NC‐EVs structural integrity was maintained after HYase treatment (Figure S6C). We studied the effect of these purified NC‐EV preparations on the three above‐mentioned pathways. NC‐EV associated effects were interpreted by comparing outcomes to the medium control processed using the same methodology. It should be noted that the dEV control was prepared by collecting the non‐EV fraction from the first density gradient, re‐running it on a second density gradient and subjecting it to the same subsequent isolation steps as the NC‐EV fraction, therefore this control only indicates whether other co‐isolated factors contribute to the observed signals, rather than confirming EV‐specificity. Furthermore, we did not perform a direct comparison between EVs and HYase‐EVs, because HYase was added before NC‐EV isolation and could affect the isolation efficiency of NC‐EVs and co‐isolates. Therefore, we conducted our statistical analysis for each specific EV isolation separately. Even though we pooled NC‐EVs from three biological replicates to obtain a representative OptiPrep purified NC‐EV preparation, NC–EV associated effects should be interpreted cautiously.

The OptiPrep‐isolated fraction demonstrated an EV‐specific effect on SMAD3 activation (Figure 6E; Ctrl vs. EV and Ctrl vs. HYase‐EV). Notably, EV‐depleted fractions had a more substantial stimulating effect compared to the EV‐effect on SMAD3 activation (Figure 6E; Ctrl vs. dEV and Ctrl vs. HYase‐dEV). Hence, both the EV fractions and EV‐depleted fractions can activate SMAD3 signalling. This was in line with the observations on the NC‐EV preparations isolated with SEC using the 5 individual spines (**Figures** 3–5). A dose‐dependent trend was observed on SMAD3 signalling based on the ratio between NC‐EVs and their corresponding EV‐depleted control (Figure 6E; serial dilution panels). Notably, with HYase treatment, the difference between NC‐EV and EV‐depleted cells was more pronounced, as evidenced by the differential activation in serial dilution steps (Figure 6E; serial dilution panels). Again, no effect on the TCF/LEF activation was observed (Figure 6F), indicating that the NC‐EVs do not activate WNT signalling in SW1353 cells. Lastly, we observed that EV‐depleted samples did not modify NF‐κB signalling in the presence of IL‐1β (Figure 6G; Ctrl IL‐1β vs. dEV and Ctrl IL‐1β vs. HYase‐dEV), whereas EVs significantly reduced NF‐κB activation (Figure 6G), Notably, HYase treatment attenuated the difference between NC‐EV and EV‐depleted controls. Overall, these results suggest that both intrinsic NC‐EV proteins as well as proteins in the soft protein corona contribute to modulation of the SMAD3 and NF‐κB signalling.

## 8 Discussion

This study expands the current knowledge of the mode of action of NC‐EVs on specific pathways involved in regenerative and anti‐catabolic processes of cartilage and the IVD. We provided a detailed atlas of the NC‐EV proteome. Whilst there was considerable overlap between NC‐EV proteins and proteins reported in Vesiclepedia, this study also revealed 166 proteins not listed in the Vesiclepedia database (version 6.1.25), including the NC‐specific marker TBXT. In addition, this study highlighted that matrisome proteins associate with the NC‐EVs and influence their mode of action. Disruption of the soft biomolecular corona, using hyaluronidase, influenced the magnitude of SMAD3 pathway activation and NF‐κB inhibition in a signalling‐specific manner. Canonical WNT was not activated regardless of the NC‐EV formulation and modification of the biomolecular corona. Overall, these data indicate that the biomolecular corona contributes to regenerative and anti‐catabolic properties of NC‐EVs.

The present study demonstrated that many NC‐EV proteins are associated with pathways involved in anabolism (SBE and TCF/LEF) and anti‐catabolism (NF‐ĸB). Functional validation in SW1353 cells demonstrated that collectively the NC‐EV proteome activated SBE signalling and inactivated IL‐1β‐induced NF‐κB signalling. The fact that NC‐EVs isolated with differential centrifugation and SEC did not functionally differ from their respective EV‐depleted controls should not be interpreted as evidence that these effects are NC‐EV independent. EVs can function as molecular hubs attracting and enhancing protein interactions and thereby elicit specific biological responses (Chrzanowski and Wolfram, 2026). It is likely that the intrinsic‐EV effects are masked by the robust signalling capacity of the matrisome‐derived biomolecular corona. These effects are dose‐dependent and a higher concentration of NC‐EVs (5*10^4^ particles per cell; undiluted) resulted in a stronger effect, highlighting the importance of dose‐optimization for clinical translation. In contrast, no effect on TCF/LEF was observed upon NC‐EV incubation for six hours using the SW1353 cells, despite the presence of multiple WNT‐related proteins in the NC‐EV proteome. These results highlight that pathway annotation does not necessarily predict functional activation in this model under the tested conditions. Altogether, this implies that NC‐EVs exert their effects at least partially via activation of SMAD3 and inhibition of the NF‐κB pathways, and not through activation of the canonical WNT signalling pathway. Whether NC‐EVs have the ability to dampen aberrantly activated WNT signalling commonly present in the degenerative process (Gill et al., 2023; Wu et al., 2021), remains to be determined. These results indicate a mode of action underlying the functional outcomes reported in dog and human pellet cultures of degenerate NP cells, showing that NC‐EVs augment GAG‐rich matrix deposition (Bach et al., 2017; van Maanen et al., 2025)_._ Furthermore, it is plausible that the observed NF‐κB inhibition may have mediated the reduction of the catabolic mediators IL‐6 and CXCL1 in human patient‐derived NP tissue stimulated with IL‐1β (van Maanen et al., 2025).

The xenogeneic approach, in which porcine NC‐EVs were studied on human cells, is a potential limitation due to possible species incompatibility and altered biological activity. Besides, it limits direct clinical translation due to potential safety and immunological concerns associated with porcine material (Denner, 2022). Previously, we have shown that porcine NC‐EVs display anabolic effects on dog and human 3D pellet cultures (Bach et al., 2015). Furthermore, we confirmed that pig NC‐EVs can bind to the human SW1353 cells. Nonetheless, the observed biologic effects may overestimate, or even more plausible, underestimate effects in a syngeneic approach. The latter comes with considerable challenges, as human NCs are only present within embryos and juvenile IVDs and their use comes with an ethical burden and technical challenges related to their limited availability. Beyond this, pathway activation and inactivation were assessed only at 6 h after stimulation as previously reported and validated (Neefjes et al., 2021). While this provides an indication of the biological response of NC‐EVs, future studies incorporating multiple timepoints could give additional insights into the signalling dynamics.

Our study underscored the important contribution of the NC‐EV biomolecular corona. Studying the biomolecular corona remains a challenge in the EV field, since loosely bound biomolecules might be disturbed based on the purification method used, restricting analysis to the analytically accessible protein corona. Therefore, different isolation methods were used to determine the function of the biomolecular corona for the targeted molecular signalling pathways (Miceli et al., 2024; Thery et al., 2018; Welsh et al., 2024). In the present study, HYase pretreatment was used to modify the GAG‐rich biomolecular corona and partly eliminate GAGs associated to NC‐EVs, as fewer GAGs per particle were detected after HYase treatment. Comparisons among different NC‐EV preparations (SEC, OptiPrep, HYase treatment) showed a distinct effect of the EV‐depleted sample on both SBE activation and NF‐κB inhibition. The effect of the biomolecular corona was more pronounced on SBE activation, and less prominent on the NF‐κB pathway, highlighting the importance of appropriate methodological controls, such as EV‐depleted controls, for data interpretation. Noteworthy, different EV‐depleted controls were used for SEC versus OptiPrep isolated EVs. SEC EV‐depleted controls were obtained by depleting EVs from the same EV‐enriched fractions using ultracentrifugation, whereas OptiPrep EV‐depleted controls were prepared by isolating the non‐EV fraction from the first density gradient and subjecting it to the same subsequent isolation steps as the EV‐fraction, as such this control should not be used to interpret EV‐unspecific signalling. Overall, our results show that the biomolecular corona has a pronounced effect in NC‐EVs, which is in line with a previous study showing that the EV cargo has a less prominent effect on the recipient cells compared to EV surface proteins (Hagey et al., 2023), lending further support to this emerging concept.

How NC‐EV surface proteins adsorb to NC‐EVs, whether dynamic rearrangement of the protein corona depends on the microenvironment, and how NC‐EVs interact with target cells in the context of the disease microenvironment warrants further exploration. To improve the understanding of how the protein corona composition contributes to their biological function, studies with variable ECM‐remodelling enzymes, beyond hyaluronidase, together with proteomic analysis of EV‐depleted samples, are required. Beyond NC‐EVs, proteomic profiling of EVs in the context of osteoarthritis (Clarke et al., 2024; Zhang et al., 2022) and disc degeneration (Li et al., 2024), demonstrates that the proteomic landscape of EVs undergoes composition changes during degeneration. While these changes are being exploited towards disease biomarkers, functional pathway analysis also links the EV‐enriched molecular pathways with the development and perpetuation of the disease.

In conclusion, this study described the proteome of purified NC‐EVs. The proteins detected in NC‐EVs included many of the classical EV marker proteins, demonstrating that the combined isolation procedure of differential centrifugation, SEC, and a sucrose density gradient could be successfully used for the isolation of these EVs. It has become clear that both the intrinsic EV protein composition and the protein corona of the NC‐EVs can modulate specific cellular signalling pathways. A better understanding of the NC‐EV proteome will likely illuminate new investigations to further elucidate the therapeutic regenerative potential of EVs on both IVD and cartilage tissues.

## Author Contributions

**Josette C. van Maanen**: conceptualization, methodology, investigation, data curation, Writing – original draft, Writing – review and editing. **Chantal Voskamp**: conceptualization, methodology, investigation, data curation, formal analysis, writing – original draft, writing – review and editing, funding acquisition. **Frank M. Riemers**: data curation, writing – review and editing. **Harmjan R. Vos**: methodology, data curation, resources, writing – review and editing. **Anouk Eversdijk**: investigation, writing – review and editing. **Marije Kleinjan**: methodology, investigation, writing – review and editing. **Menno Bergmeijer**: methodology, investigation, data curation, writing – review and editing. **Martijn J. C. van Herwijnen**: writing – review and editing. **Stuart C. Howes**: methodology, data curation, resources, writing – review and editing. **Frances C. Bach**: conceptualization, methodology, investigation, writing – review and editing. **Tim J. M. Welting**: conceptualization, methodology, data curation, resources, writing – review and editing. **Christine L. Le Maitre**: writing – review and editing, supervision, funding acquisition. **Guus G. H. van den Akker**: conceptualization, methodology, investigation, formal analysis, resources, writing – review and editing. **Marca H. M. Wauben**: conceptualization, methodology, formal analysis, resources, writing – review and editing, supervision. **Mariana A. Tryfonidou**: conceptualization, methodology, formal analysis, resources, writing – original draft, writing – review and editing, supervision, funding acquisition.

## Funding

This work was supported by Nederlandse Organisatie voor Wetenschappelijk Onderzoek, 19251 and the Horizon 2020 Framework Programme, 825925. Cryo‐electron microscopy studies in this work were supported by the Basic Research Grant 2025 of the International Society for the Study of the Lumbar Spine.

## Geolocation Information

This study was conducted at the Faculty of Veterinary Medicine, Utrecht University, Utrecht, the Netherlands; the Center for Molecular Medicine, University Medical Center Utrecht, Utrecht University, Utrecht, the Netherlands; and the Department of Orthopaedic Surgery, Maastricht University Medical Center+, Maastricht, the Netherlands.

## Disclosure

Authors have completed the ICMJE Disclosure Form.

## Ethics Statement

All animal procedures were in accordance with national regulations.

## Consent

Not applicable, as this study did not involve human participants or identifiable patient data.

## Permission to Reproduce Material

Figures 3A, 4A and 5A were created with BioRender.com. The authors have obtained the appropriate license for publication.

## Conflicts of Interest

The authors declare conflicts of interest.

## Supporting information




**Supporting information**: jex270186‐sup‐0001‐SuppMat.docx

**Supporting information**: jex270186‐sup‐0002‐SuppMat.xlsx

**Supporting information**: jex270186‐sup‐0003‐SuppMat.xlsx

## Data Availability

The mass spectrometry proteomics data have been deposited to the ProteomeXchange Consortium via the PRIDE (Perez‐Riverol et al., 2025) partner repository with the dataset identifier PXD072052.
