# Supplementary material for: Proteomic Atlas of Notochordal Cell‐Derived Extracellular Vesicles Highlights EV‐Specific NF‐κB Modulation and Functional Implications of the Protein Corona
Source: J Extracell Biol. 2026 Sep 13;5(9):e70186. doi: 10.1002/jex2.70186 (PMC13572908; doi:10.1002/jex2.70186)
Supplement: Supplementary file 1 [file JEX2-5-e70186-s001.docx]

**SUPPLEMENTARY INFORMATION**

**SUPPLEMENTARY MATERIALS AND METHODS DETAILS**

**Isolation of NC-EVs from conditioned media**

NCCM was generated in two batches (pig 1-2 and 3-7). Intervertebral discs were opened and NC-rich NP tissue collected under sterile conditions. Conditioned media was generated by culturing NC rich NP tissue for 4 days (1 gram tissue/20 mL media) in high glucose (Hg)DMEM (Gibco, 31966) supplemented with 1% penicillin/streptomycin (P/S, Gibco, 15140122) at 37 ˚C, 5% CO_2_ and 5% O_2_. After 4 days, the NCCM was filtered through a 100 µm cell strainer to remove NP tissue. NCCM was collected and processed for isolation of NC-EVs for mass spectrometry and functional cell culture studies. NCCM was stored for maximal 2 months for proteomic analysis and maximal 3.5 years for functional analysis, and used after one freeze-thaw cycle. Each pig spine served as a biological replicate in the down-stream studies and analysis.

*Size exclusion chromatography (SEC) with commercial columns for functional studies*

qEV SEC-columns (iZON Science, 1 mL sample/column) were equilibrated using

PBS (Gibco, 10010023) for mass spectrometry or with high glucose (Hg)DMEM (Gibco, 31966) for targeted functional studies. For each mL of 5xNCCM, twenty-five fractions of 0.5 mL were collected per qEV column. The protein concentration of each fraction was determined at 280 nm (DeNovix, DS-11). Based on the characterization of EV markers of the SEC fractions in previous work^17^, and the measured protein concentrations (**Figure S1**), the six fractions containing NC-EVs (between fractions 7-13) were pooled per pig spine. To generate the EV-depleted samples, the EV-enriched samples were depleted from EVs by ultracentrifugation at 100,000*g* (Beckman Coulter Optima XE-90, SW60 rotor, RCF average 100,848 *g*, RCF max 131,921 *g*) for 15 - 18 hours at 4˚C, whereafter the supernatant was collected. All NC-EV and EV-depleted samples were stored in low protein-binding tubes until further use in culture experiments at -80 ˚C.

*SEC and sucrose density gradient centrifugation for mass spectrometry*

After SEC, the EV-enriched samples derived from 6 SEC columns per biological replicate were pooled (yielding 18 mL and corresponding to ~1.5 gram of NP tissue). The pooled samples were topped up with PBS in SW41 tubes (13.2 mL, Beckman Coulter Optima XE-90) and centrifuged at 100,000*g* for 65 minutes (4 ˚C, SW41 rotor, 28,500 rpm, RFC average 100,069*g*, RFC max 139,187). The 100,000*g* pellets of the pooled EV fractions were resuspended in 50 µL PBS per donor and stored in low protein-binding tubes at -80 ˚C until further use. Further purification of the EV-enriched fraction for mass spectrometry analysis was achieved with linear sucrose density gradients. The 50 µL EV-enriched fraction/animal was resuspended in 1.5 mL 2.5 M sucrose and loaded into a SW41 tube. The gradient was built by loading 15 successive sucrose fractions of 700 µL (ranging from 2.0 M to 0.4 M sucrose, with a decrement of 0.114 M per fraction) on top of the sample. The gradients were centrifuged at 192,000*g* (Beckman Coulter XE-90, 15 - 18 hours, 4˚C, SW41 rotor, 39,500 rpm, RFC average 192,223*g*, RFC max 267,364*g*). After ultra-centrifugation, fractions of 500 µL were collected from the top (total of 25 fractions/gradient). The density of the collected fractions was measured by refractometry to identify the fractions containing NC-EVs (densities of 1.09–1.16 g/mL^10^). The EV-containing fractions were pooled per pig spine, diluted with PBS and pelleted by ultracentrifugation at 100,000*g* (Beckman Coulter Optima XE-90, 65 minutes, 4˚C, SW32 rotor, 28,500 rpm, RFC average 100,069*g*, RFC max 139,187*g*). The 100,000*g* pellets of the pooled-EV fractions were resuspended in 10-15 µL PBS per pig spine (NC-EV sample) and stored in low protein binding 1.5 mL tubes at -80˚C until mass spectrometry was performed.

*Iodixanol density gradient centrifugation and SEC for validation functional studies*

Due to limited sample availability, this set of experiments was performed using a pooled sample: differentially centrifuged conditioned media (5xNCCM) from three pig spines were pooled, yielding a total volume of 13 mL. The 13 mL was divided equally, with 6.5 mL undergoing hyaluronidase treatment^18^, to hydrolyze GAGs from the NC-EV surface, and 6.5 mL was left untreated. Each 6.5 mL was loaded on a 60-10% iodixanol gradient (Optiprep, Progen Biotechnik GmbH) in an SW40 tube. The gradient was built by loading 15 successive iodixanol fractions of 350 µL (ranging from 60% to 10% iodixanol, with a decrement of 2.9% per fraction). Optiprep gradients were ultracentrifuged at 39,000 rpm (Beckman Coulter Optima XPN-80 with a SW40Ti rotor, RCF average 192,072, RCF max 270,519 *g*) for 15–18h at 4 ˚C. Fractions of 500 µL and densities of 1.06-1.19 g/mL were collected and pooled. To generate a NC-EV depleted methodological control, 6.5 mL left on top of the gradient was collected and loading onto a new Optiprep gradient and the same fractions were collected (**Figure 6A**). To replace Optiprep from the NC-EV preparation with HgDMEM for functional analysis, subsequently a SEC column (20 mL; Bio-Rad Laboratories) with 15 mL Sephadex g 100 (Sigma-Aldrich) was used and 24 fractions of 1 mL were collected. The six fractions containing NC-EVs (between fractions 3-9) were pooled and stored in low protein-binding tubes until further use in culture experiments at -80˚C.

**Liquid chromatography-tandem mass spectrometry (LC-MS/MS)**

All samples were digested with trypsin: 5-7.5 µL of sample was lyophilized using a speedVac, after which 60μL lysis/alkylation buffer (8 M urea, 1 M ammonium bicarbonate (ABC), 10mM TCEP (tris (2-carboxyethyl) phosphine hydrochloride), 40 mM 2-chloro-acetamide) was added. After vortexing and a short spin, samples were placed in a sonication bath for 30 min, after which the urea concentration was reduced 4-fold with 180 µl 50 mM ABC + 250 ng trypsin/LysC mix (V5071, Promega). After overnight digestion, peptides were loaded on homemade C-18 stage tips (3 M, St Paul, MN) (4 layers), eluted with 80% acetonitrile and, after evaporation of the solvent in the speedvac, redissolved in 0.1% formic acid. After separation on a 25 cm in-house pulled micro tip-column (50 µm ID) in-house packed with C-18 material (1.9 µm aquapur gold, dr. Maisch) using a 240-minute gradient (5% to 80% acetonitrile, 0.1% formic acid), delivered by an easy-nLC 1200 (LC140, Thermo Scientific), peptides were electro-sprayed directly into a Orbitrap Eclipse Mass Spectrometer (FSN04-10000, Thermo Scientific). The latter was set in data dependent mode with a cycle time of 1 second, in which the full scan over the 400-1200 mass range was performed at a resolution of 240,000 and the High field asymmetric waveform ion mobility spectrometry (FAIMS) switched between -45 and -65 compensation voltage. The most intense ions (intensity threshold of 10,000 ions) were isolated by the quadrupole where after they were fragmented with a HCD collision energy of 30%. Fragments were detected in the iontrap in rapid mode with a maximum injection time set to 40 milliseconds.

**Data analysis of mass spectrometry data**

Raw data were analyzed with Proteome Discoverer 2.5 (Thermo scientific), using the Uniprot database for *Sus Scrofa* (taxonomy ID: 9823), extracted from UniprotKB on December 2023. All proteins that failed to be assigned to a protein name, but did receive an accession number, were manually annotated with UniprotKB December 2023. The resulting protein abundance data were imported into Perseus (v2.10.0)^19^. The groups and samples were annotated, and only proteins identified with at least 3 peptides of which 2 were unique peptides, were further analyzed. In case of multiple variants, the corresponding values were summed. All values were log2 transformed, and for differential analysis only proteins recovered in all four of seven biological replicate EV samples were used for further analysis.

*Enrichment and pathway analysis*

Enrichment analysis on the proteins which were identified in at least four NC-EVs samples was done using G:GOSt (g:Profiler version e112_eg59_p19_25aa4782)^25^. As data source, Gene Ontology for biological processes and Reactome for biological pathways was selected, using a term size between 5-350 proteins to select for specific terms. The identified terms were considered significant if the *p*-value was lower than 0.05 (*p*-value was corrected for multiple testing within each category using the Benjamini–Hochberg procedure). Next, we clustered the individual enrichment terms into different categories based on functional overlap (**Table S1**). Key proteins were identified using pathway databased in KEGG (TGFβ map04350, WNT map04310, NF-κB map04064 and JAK-STAT map04630).

*Comparing NC-EV proteome to other EVs*

The Vesiclepedia database (version 6.1.25) was used to compare the NC-EV proteome with the proteome of other EVs^26^. For this, proteins that were identified in at least four NC-EVs samples were used. Results from these comparisons were visually represented in Venn diagrams created with the web-based tool InteractiVenn^27^

**Nanoparticle tracking analysis (NTA)**

The concentration and size of the NC-EVs and EV-depleted samples were determined using NTA with a NanoSight NS500 (Malvern Panalytical, Eindhoven, the Netherlands) containing a sCMOS camera and 405 nm laser. The measurements were performed with NTA 3.4 software build 3.4.4 for data acquisition and processing (Malvern Panalytical, Eindhoven, the Netherlands). Each sample was diluted in Dulbecco's Phosphate Buffered Saline (DPBS, Sigma‐Aldrich, D8537; 1:50 NC-EVs and 1:5 EV-depleted samples). For each sample, five captures of 30 s each were acquired with the temperature controlled at 25°C and with the viscosity set to that of water (0.9 cP). Samples were captured at camera level 16 with a shutter of 1,300 and gain of 512 (default settings). Images were processed with a detection threshold of 20 and automatic blur.

**EV depletion of reagents containing fetal bovine serum**

For the generation of EV‐depleted fetal bovine serum (FBS, Gibco, 105000-64) was centrifuged at 100,000 *g* (Beckman Coulter Optima XE-90, SW32, RCF average 100,069*g,* RCF max 139,187 g) for 15–18 h at 4°C. The supernatant was collected and used as EV-depleted FBS.

**NC-EV:SW1353 cellular association**

*NC-EV labeling* *with Malemide-488 and MemGlow-640*

NC-EVs were purified from 5x NCCM using SEC as previously described. Six fractions between 7-13 and samples from three pig spines (pig 1,5,6) were pooled, yielding a total volume of 9 mL in PBS. To generate EV-depleted controls, 4 mL of the EV-enriched samples were depleted from EVs by ultracentrifugation at 31,200 (Beckman Coulter Optima XE-90, SW60 rotor, RCF average 100,848 *g*, RCF max 131,921 *g*) for 15-18 hours at 4 ˚C, whereafter the supernatant was collected. Both the EV-enriched (4 mL) and the EV-depleted samples (4 mL) were 20 times concentrated using Amicon Ultra-15 centrifugal filters 100K (Merck) at 3,214*g* and 4˚C for the required time, until approximately 150 µL of PBS was left in the filter (approximately 8 hours). Concentrated samples were stored in low protein binding tubes until further use at -80 ˚C. To each 50 µL of the EV-enriched, EV-depleted or PBS control, 5 µL of C5-maleimide-Alexa 488 (Thermo Fisher Scientific; used at a final concentration of 20 µg/mL) was added for 2 h at RT, or 200 µL MemGlow 640 (Cytoskeleton; used at a final concentration of 400 nM) was added for 30 min at RT. To remove the EV-labeling dye, samples were washed using SEC columns (iZON Science, 1 mL sample/column) using HgDMEM as previously described. After labelling samples were analyzed on the CytoFLEXnano (Beckman Coulter) to determine their percentage of fluorescent labeling.

*NC-EV:SW1353 interaction*

To determine whether porcine NC-EVs could interact with human SW1353, cellular association was studied with labeled NC-EVs. SW1353 cells (ATCC; HTB-94) were thawed and expanded for at least two passages in DMEM/F12 (31331028, Gibco) supplemented with 10% FBS. The cells were trypsinized and reseeded (30,000 cells/well into 96-well plates and serum-starved overnight in HgDMEM supplemented with 0.5% EV-depleted FBS, 1% P/S and 0.5% Fungizone. After overnight starvation, the cells were washed twice with 100 µL PBS and stimulated for six hours with NC-EV labeling Malemide-488 or MemGlow-640 stained NC-EVs or their respective controls. After stimulation, the cells were washed once with 100 µL PBS, after which they were trypsinized and analyzed using the Cytoflex S Flow Cytometer (Beckman Coulter).

**Functional pathway validation using stable reporter cell lines**

To validate specific pathways that may be influenced by NC-EVs, three reporter cell lines were used based on SW1353 cells (ATCC; HTB-94) generated by lentiviral transduction as described previously^15^. Here, we implemented lines reporting the activity of consensus binding sequences specific for the transcription factors of NF-ĸB, Smad binding element (SBE), and TCF/LEF (**Table S2**). SW1353 reporter cell lines were thawed and expanded for at least two passages in DMEM/F12 (31331028, Gibco) supplemented with 10% FBS and 1% penicillin–streptomycin (15240062, Gibco).

All reporter cell lines were stimulated with hgDMEM control medium, or hgDMEM control medium that was subjected to the same isolation procedure as NC-EVs derived after SEC isolation, in the present or absence of the positive stimuli (**Table S2**). We observed significant functional differences in the presence of SEC control medium compared to control medium (**Figure S7**), and therefore SEC control medium was used for further analysis.

**SUPPLEMENTARY FIGURES**

**Figure S1. Overview of different NC-EV isolation methods**

NC-EVs were enriched by differential centrifugation, size exclusion chromatography (SEC) and sucrose density gradient-based purification methods, to minimize the number of non-EV particles inevitably co-isolated. For functional analysis NC-EVs were only enriched by differential centrifugation in combination with SEC to avoid functional loss of proteins. To confirm NC-EV specific functional effects EV-depleted controls were included as controls. In addition, we repeated key functional experiments using a pool of biological replicates with a more stringent purification method using OptiPrep-based purification method in combination with differential centrifugation and SEC to maintain functionality and increase purity. To this end, Opti-Prep enriched EV formulations were subjected to hyaluronidase treatment prior to purification to strip away part of the GAG-rich biomolecular corona.

**Figure S2. Proteomic analysis of NC-EV from seven porcine donors**

A. Per pig spine six size exclusion chromatography columns were used (no. 1-6) to obtain 18 mL of pooled EV-enriched medium. Twenty five fractions (or fifteen for the first two donors) were collected and the protein concentration was measured at 280 nm. Six fractions, starting from the start of the first protein peak, were collected (between fraction 7-13, indicated in green) as these were expected to contain the most EVs. As the start of the protein peak and the rest of the proteins profile were consistent between pig 4-7, for pig 1 and 2, only fractions 1-15 were measured. Unfortunately, the protein measurement data for pig 3 were lost due to a technical malfunction. B. NanoSight measurements show particles size distribution of EV-depleted controls after size exclusion chromatography. Event detection was lower compared to EV related samples (2-6 particles/frame). Graphs shows mean and SD (red) of five captures of 30 seconds. C. Number of unique proteins that were identified for each porcine donor and shared among donors. D. Heat map of all the NC-EV proteins identified. Missing data imputed with a constant value of 10.

**Figure S3. Identified NC-EV proteins that have not been reported in Vesiclepedia**

Venn diagram shows overlap between NC-EV proteome and proteins in Vesiclepedia database (version 6.1.25).

**Figure S4. Pathway analysis**

Schematic representation of the pathway analysis workflow for proteins identified in NC-EV in at least four biological replicates around the selected signaling pathways (NFκB, TGFβ and WNT).

**Figure S5. NC-EVs interact with SW1353 cells**

Quantification of data obtained by flow cytometry. SW1353 cells were cultured for 6 hours with either MemGlow-labeled NC-EVs or Maleimid-labeled NC-EVs. As a control SW1353 cells were cultured with MemGlow- or Maleimid-labeled NC-EV depleted (dEV) or with MemGlow- or Maleimid-labeled labeled PBS. Plots show percentage of positive cells (A) or mean fluorescence intensity (MFI B-C). Bars represent mean ± standard deviations and each dot represents a technical replicate. Samples from three pig donors (1, 5 and 6) were pooled and used for analysis.

**Figure S6. Characteristics of NC-EVs that are isolated with optiprep, size exclusion chromatography, and hyaluronidase**

A. Size distrubtion of optiprep isolated NC-EVs and their respective depleted control using ZetaView analysis. B. Concentration of glycosaminoglycans (GAGs) in µg per 10^6^ particles. C. Cryo-electron microscopy images showing NC-EV structural integrity. Scale bar represents 200 nm.

**Figure S7. Size exclusion chromatography fractionates high glucose DMEM and interferes with reporter activity**

A-C. Luciferase activity of SBE (A), TCF/LEF(B), and NFκB (C) reporters in SW1353 cells. Cells were either cultured with or without stimulators (TGFβ3 for SBE; WNT3A for TCF/LEF; IL1β for NFκB) in standard high glucose DMEM (hgDMEM) media or in hgDMEM media that was processed through size exclusion chromatography, with fractions 7-13 collected and pooled before use. All media were supplemented with 0.1% EV-depleted FBS and 1% P/S to avoid interference from FBS-derived factors with NC-EVs and to allow for showcasing EV-mediated functional effects. Bars represent mean ± standard deviations and each dot represents a technical replicate. Brackets indicate one-way ANOVA tests’ significance.

**SUPPLEMENTARY TABLES**

**Table S1. NC-EV proteome enrichment terms**

| Overall enrichment term | Number of terms |
| --- | --- |
| Vesicle-mediated transport | 161 |
| Metabolic processes | 118 |
| Signal transduction | 99 |
| (Intra-)cellular transport | 95 |
| Cell differentiation | 70 |
| Cellular response to stimuli | 70 |
| Cytoskeleton organization | 61 |
| Regulation of cell cycle and growth | 54 |
| Regulation of molecular function | 56 |
| Translation | 53 |
| Tissue remodeling | 44 |
| Regulation of protein degradation | 40 |
| Extracellular matrix organization | 36 |
| Diseases | 35 |
| Regulation of cell death | 33 |
| Cell adhesion | 30 |
| Migration | 30 |
| Protein localization to membrane | 26 |
| Regulation of GTPase activity | 21 |
| RNA processing | 15 |
| Cell surface organization | 13 |
| Cell polarity | 9 |

**Table S2. Overview of the response element driven nano luciferase reporters as previously published by Housmans *et al.* Osteoarthritis Cartilage. 2023**

| Reporter | Stimuli | Recognition sequence | Transcription factor | Upstream activator |
| --- | --- | --- | --- | --- |
| SBE | 10 ng/mL TGFβ3 | AGTATGTCTAGACTGAAGTATGTCTAGACTGAAGTATGTCTAGACTGA | SMAD3;SMAD4 | TGFβ/ALK5 |
| TCF/LEF | 200 ng/mL WNT3A | CCTAAGATCAAAGGGACTAAGATCAAAGGGTTTAAGATCAAAGGGCTTAAGATCAAAGGGCCTA | TCF:LEF | WNT/β-catenin |
| NFĸB | 0.1 ng/mL IL1β | GGGAATTTCCGGGGACTTTCCGGGAATTTCCGGGGACTTTCCGGGAATTTCC | NFĸB | IL1β/IKK/IĸB |

**Supplementary Table 1: Excel file with NC-EV proteome**

**Supplementary Table 2: Excel file with enrichment terms**
